# Gut microbiota in patients with COVID-19 and type 2 diabetes: A culture-based method

**DOI:** 10.3389/fcimb.2023.1142578

**Published:** 2023-02-09

**Authors:** Pavlo Petakh, Nazarii Kobyliak, Aleksandr Kamyshnyi

**Affiliations:** ^1^ Department of Biochemistry and Pharmacology, Uzhhorod National University, Uzhhorod, Ukraine; ^2^ Department of Microbiology, Virology, and Immunology, I. Horbachevsky Ternopil National Medical University, Ternopil, Ukraine; ^3^ Medical Laboratory CSD, Kyiv, Ukraine; ^4^ Endocrinology Department, Bogomolets National Medical University, Kyiv, Ukraine

**Keywords:** gut microbiota, coronavirus disease 2019, dysbiosis, metformin, diabetes

## Abstract

**Background:**

The global pandemic of coronavirus disease 2019 (COVID-19) continues to affect people around the world, with one of the most frequent comorbidities being Type 2 Diabetes (T2D). Studies have suggested a link between disbalances in gut microbiota and these diseases, as well as with COVID-19, potentially due to inflammatory dysfunction. This study aims to analyze the changes in gut microbiota in COVID-19 patients with T2D using a culture-based method.

**Methods:**

The stool samples were taken from 128 patients with confirmed COVID-19. Changes in the composition of gut microbiota were analyzed by culture-based method. The study used chi-squared and t-test to find significant differences in gut bacteria between samples and non-parametric correlation analysis to examine relationship between gut bacteria abundance, C‐reactive protein (CRP) levels and length of stay (LoS) in COVID-19 patients without T2D.

**Results:**

The gut microbiota of T2D patients with COVID-19 showed increased *Clostridium* spp., *Candida* spp., and decreased *Bifidobacterium* spp.*, Lactobacillus* spp. Metformin-treated patients with T2D and COVID-19 without antibiotic treatment showed increased *Bacteroides* spp., *Lactobacillus* spp., and decreased *Enterococcus*, *Clostridium* compared to the same group with antibiotic treatment. The study also found a positive correlation between the abundance of certain gut microbiota genera, such as *Klebsiella* spp. and *Enterococcus* spp., and CRP levels and LoS in COVID-19 patients without and with T2D, while the abundance of other genera, such as *Bifidobacterium* spp. and *Lactobacillus* spp., was found to have a negative correlation.

**Conclusion:**

In conclusion, this study provides important insights into the gut microbiota composition of SARS-CoV-2-infected individuals with T2D and its potential impact on the course of the disease. The findings suggest that certain gut microbiota genera may be associated with increased CRP levels and longer hospital stays. The significance of this study lies in the fact that it highlights the potential role of gut microbiota in the progression of COVID-19 in patients with T2D, and may inform future research and treatment strategies for this patient population. The future impact of this study could include the development of targeted interventions to modulate gut microbiota in order to improve outcomes for COVID-19 patients with T2D.

## 1 Introduction

In December 2019, the first reports of unknown pneumonia cases emerged in Wuhan, China (She et al., 2020). The virus responsible for the illness was identified as a new coronavirus, which was named SARS-CoV-2, and it was found to have a genetic similarity to SARS-CoV (Hu et al., 2021). Since then, 4 years have passed and the world has experienced multiple waves of COVID-19 outbreaks caused by different versions of the SARS-CoV-2 virus, known as variants. So far, the Alpha, Beta, Gamma, Delta, and Omicron variants have been designated as “variants of concern” due to their high infectivity and virulence, and each later one appears to be more transmissible than the previous one (Shrestha et al., 2022). The pandemic is ongoing, and the dominant strain currently is Omicron.

Type 2 diabetes (T2D) is a common comorbidity among people with COVID-19, with a prevalence that ranges between 7-30% (Corrao et al., 2021). People with diabetes who are infected with SARS-CoV-2 have a higher risk of being hospitalized, developing severe pneumonia, and a higher mortality rate compared to those without diabetes (Lima-Martínez et al., 2021). The chronic elevation of blood sugar levels associated with T2D can weaken innate and adaptive immunity. Additionally, T2D is linked to a low-grade chronic inflammation state that can exacerbate the inflammatory response and increase the risk of developing acute respiratory distress syndrome (Al-Kuraishy et al., 2021; Petakh et al., 2022).

One of the factors influencing the course of COVID-19 is the gut microbiota (Petakh et al., 2022). Recent research is starting to uncover the connection between gut microbiota and the way COVID-19 affects the body. The gut, being the largest immune organ in the body, has its own set of microorganisms that can control host immunity, protect against pathogens and aid in nutrient digestion (Belkaid and Hand, 2014). Gut dysbiosis with a reduction in microbial diversity is commonly linked to immune-mediated inflammatory and autoimmune diseases (Mousa et al., 2022). The gut microbiota could also regulate local and systemic inflammatory activity, and some studies have demonstrated that respiratory infections are associated with both compositional and functional alterations of the gut microbiota through vital crosstalk between gut microorganisms and the pulmonary system, otherwise known as the “gut–lung axis” (Cruz et al., 2021). It is also interesting that gut dysbiosis is observed not only in patients with COVID-19, but also in patients with T2D and other non-communicable diseases (Iatcu et al., 2021).

Currently, molecular-based methods are used to assess the gut microbiota, such as 16S rRNA gene sequencing, but the main disadvantage of these methods is the price. In this study, we used the culture-based method, which is relatively cheap and can be used in low-income countries.

## 2 Materials and methods

### 2.1 Study design and sample collection

**Figure 1:**
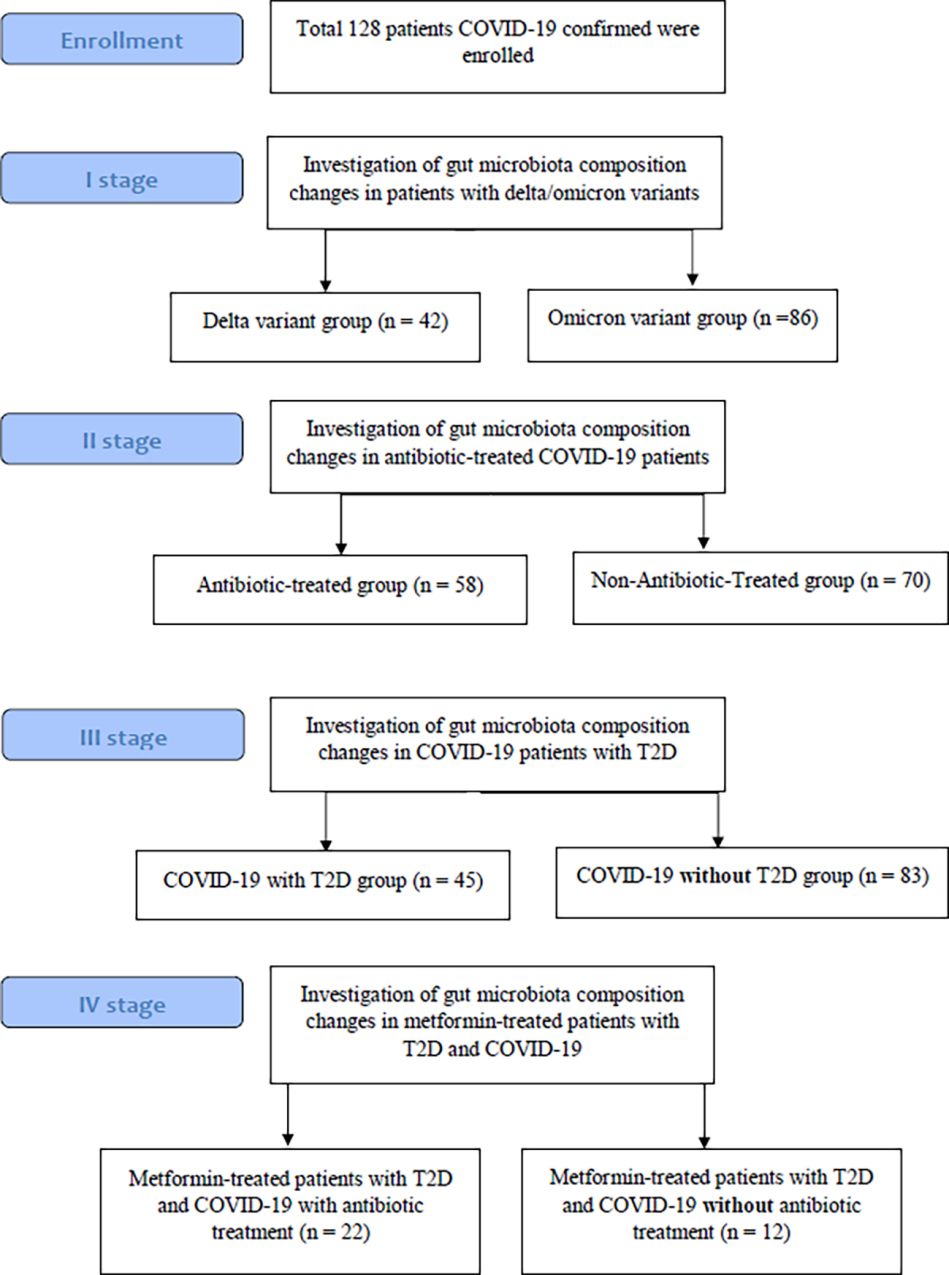
Study flow chart.

From COVID-19 confirmed patients who were admitted to the Transcarpathian Regional Infectious Hospital from 2020 to 2022, 128 feces samples were collected. The study had four stages, each of which analyzed the characteristics of the gut microbiota in different types of groups of patients with COVID-19 (**Figure 1**). Patients with COVID-19 were assigned to the Delta and Omicron groups based on the predominance of the respective strains during this period. In the group of patients who used antibiotics, 29.3% of patients were prescribed linezolid, 34.4% were prescribed meropenem, 25.8% were prescribed fluoroquinolones (moxifloxacin and ciprofloxacin), and 10.5% were prescribed cephalosporins of the III or IV generations. Metformin-Treated Patients with T2D (MTP with T2D) took metformin in a dose of 1000-1500 mg per day for at least 3 months before admission.

### 2.2 Microbiota analysis

**Figure 2:**
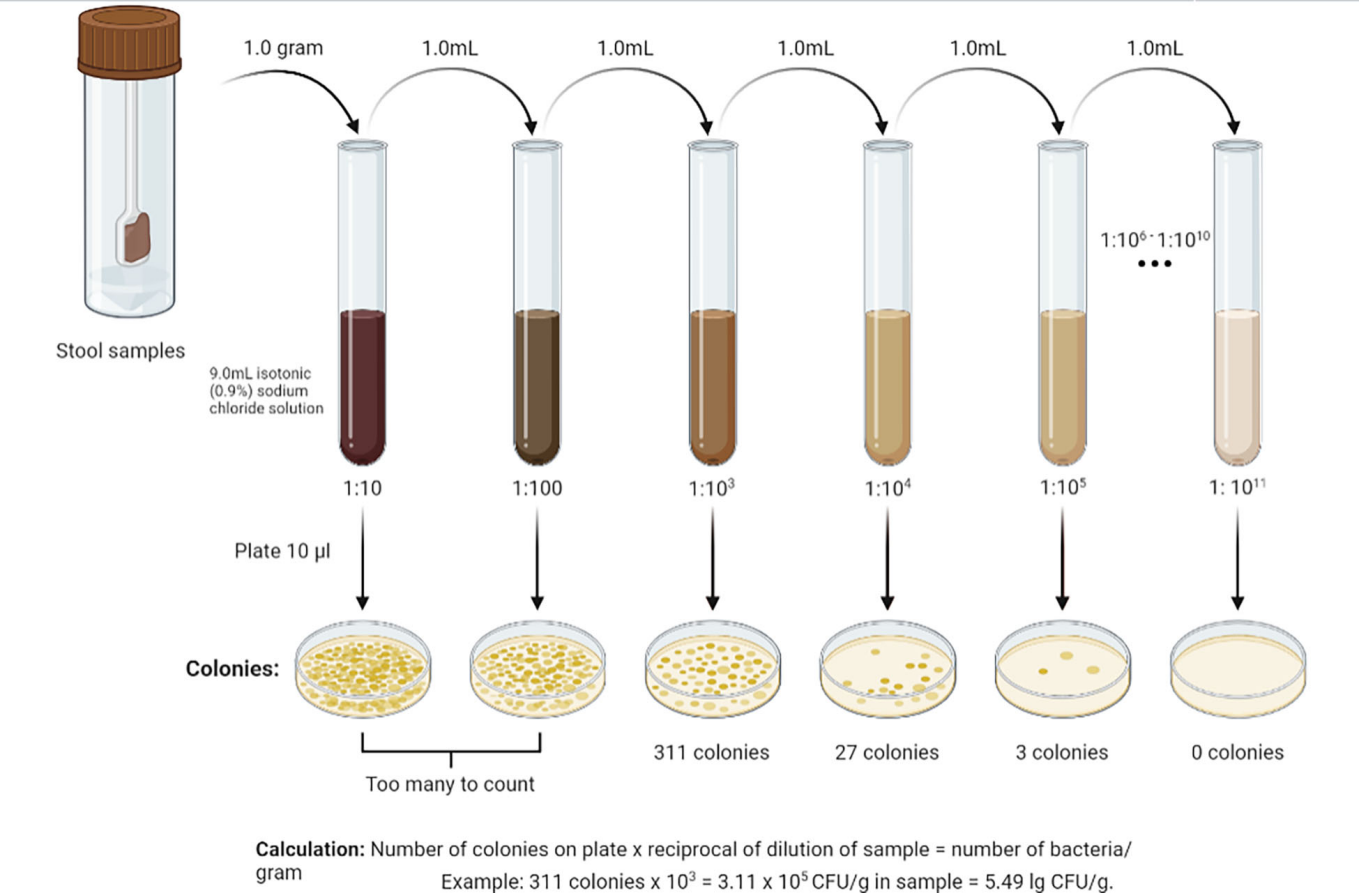
Methodology for serial dilution of stool samples.

**Figure 3:**
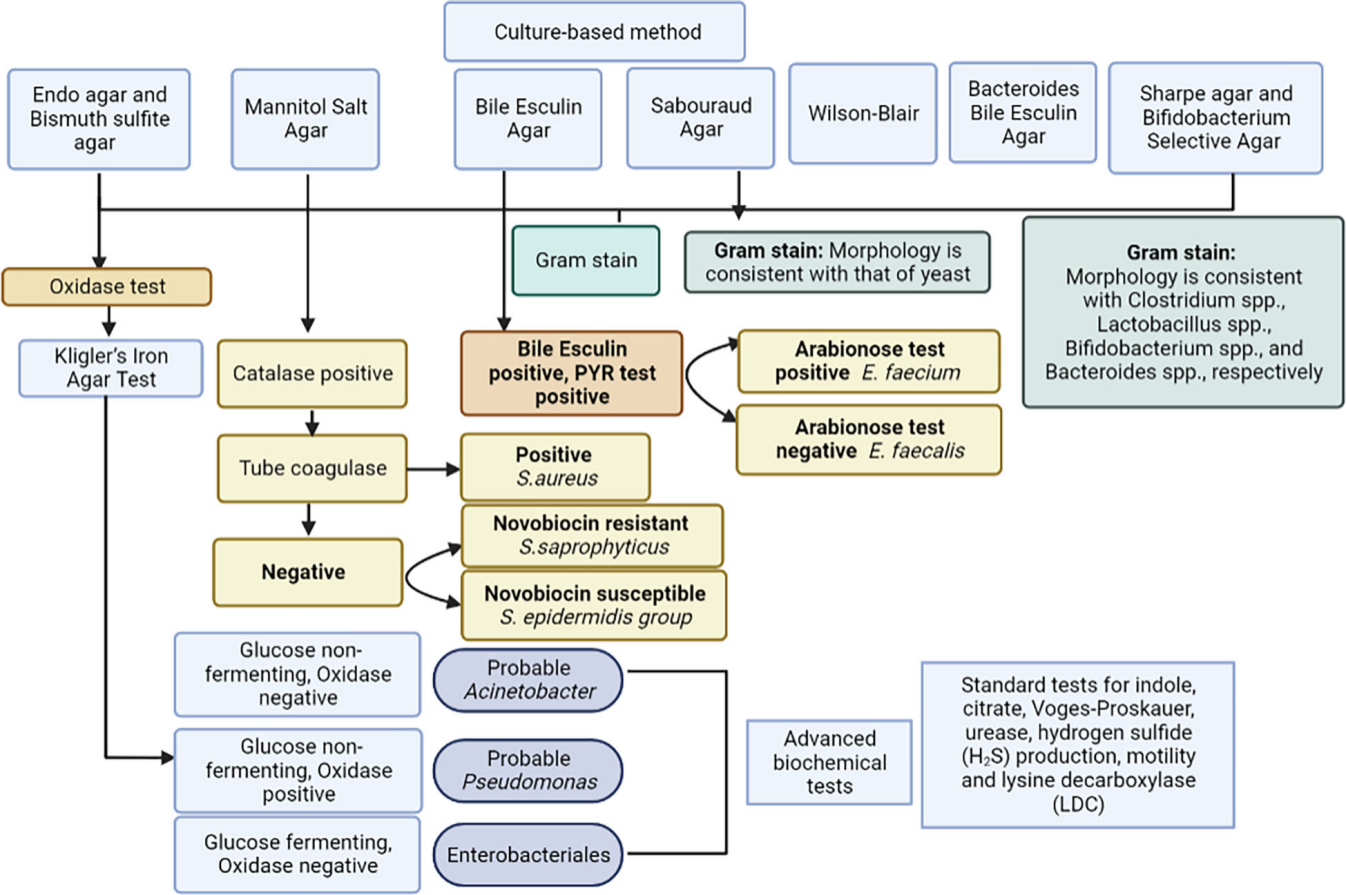
Methodology for bacterial identification.

For analysis of gut microbiota, grams of feces were weighed, 9 ml of isotonic (0.9%) sodium chloride solution were added to a test tube, and the mixture was thoroughly rubbed until a homogeneous mass was formed. This created a 10^-1^ dilution. Subsequently, a series of dilutions from 10^-2^ to 10^-11^ were prepared in the same way (**Figure 2**). Using sterile micropipettes, 10 μl was taken from each dilution and applied to nutrient media. For the isolation of enterobacteria, commercial nutrient media Endo agar and Bismuth sulfite agar were used; for *Staphylococcus* spp. - Mannitol Salt Agar; for *Enterococcus* spp. – Bile Esculin Agar; for Yeast – Sabouraud Agar. Iron sulfite agar (Wilson-Blair) was used for isolation of Clostridia, Sharpe agar for *Lactobacillus* spp., Bifidobacterium Selective Agar for *Bifidobacterium* spp., and Bacteroides Bile Esculin Agar for *Bacteroides* species. Identification of microorganisms was carried out according to the scheme (**Figure 3**) based on the Clinical Microbiology Procedures Handbook, Volume 1-3, 4th Edition (Dunn et al., 2016). For the convenience of presentation of the material and mathematical and statistical processing, decimal logarithms of the quantitative indicator of the grown colonies of microorganisms (lg CFU/g) were used, and the proportion of genera were also calculated.

### 2.3 Statistical analysis

To determine whether any differences observed between samples were statistically significant, we used the chi-squared test, which compares the observed frequencies of different bacterial taxa to the expected frequencies. We also used the Student’s t-test, which compares the mean values of different bacterial taxa between two groups of samples. In the current study, non-parametric correlation analysis was also used to examine the relationship between the abundance of various bacterial genera and C‐reactive protein (CRP) levels and length of stay (LoS) in COVID-19 patients without T2D. Spearman’s rank correlation coefficient (r) was calculated to determine the strength and direction of the correlation, with a p-value of <0.05 considered statistically significant. All statistical analyses were performed using the IBM SPSS Statistics and GraphPad Prism 9, and a p-value of <0.05 was considered statistically significant.

## 3 Results

**Figure 4:**
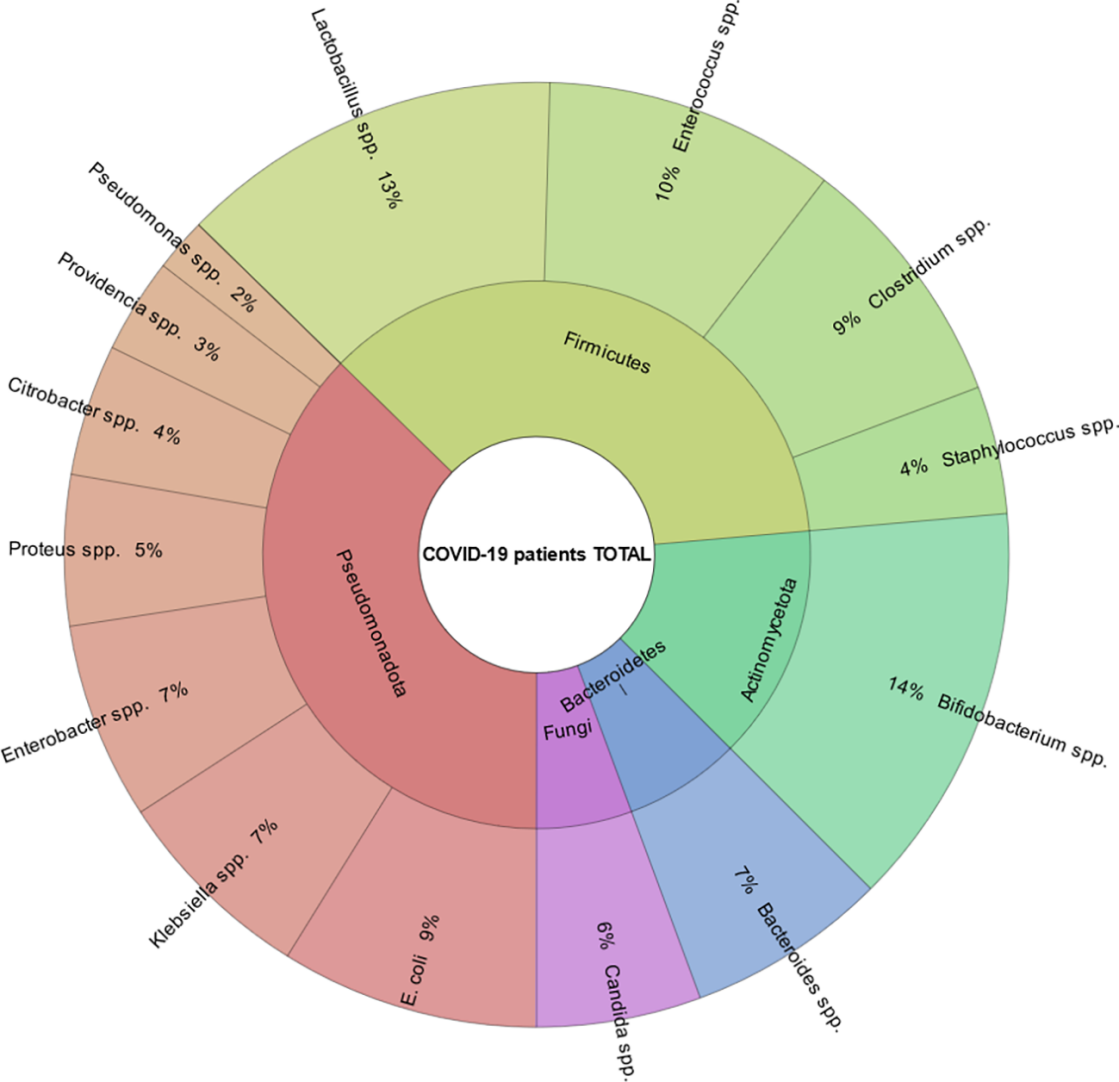
Median percentage of gut bacteria in all patients included in the study.

**Figure 5:**
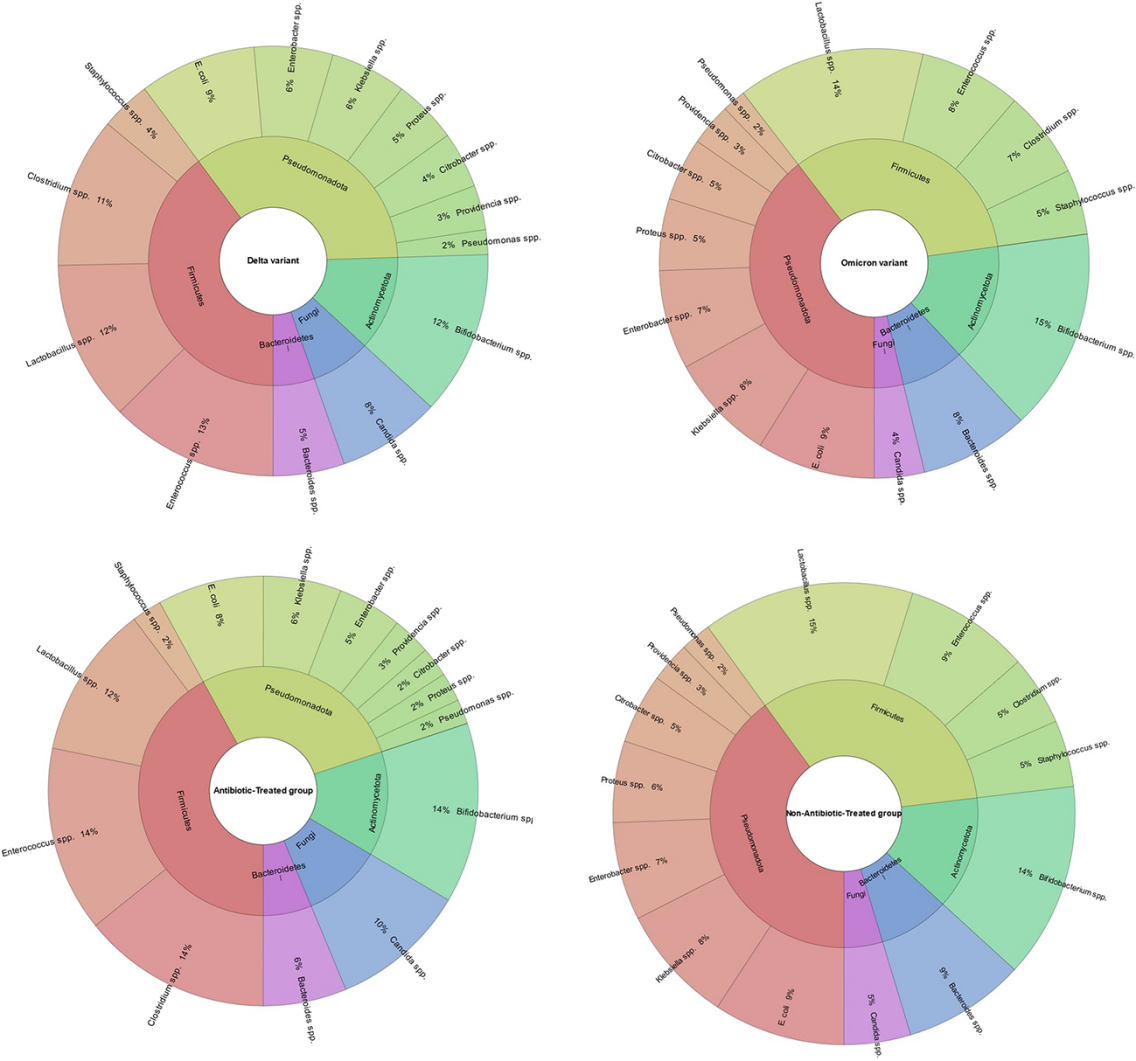
Comparison of gut microbiota composition in patients infected with Omicron or Delta variants of SARS-CoV-2 and Antibiotic-Treated or Non-Antibiotic-Treated group. The results indicate that the Omicron group had a significantly higher abundance of the genera *Bifidobacterium, Bacteroides, Klebsiella, Enterobacter*, and *Lactobacillus* compared to the Delta group (P<0.05). In contrast, the Delta group had a significantly higher abundance of the genera *Clostridium, Enterococcus, and Candida* compared to the Omicron group (P<0.05). The figure also shows the comparison of gut microbiome composition between patients treated with antibiotics and those who were not. It demonstrates that patients treated with antibiotics had a higher abundance of the genera *Enterococcus, Candida*, and *Clostridium*, while non-antibiotic-treated group had a higher abundance of the genera *Proteus, Klebsiella, Citrobacter, Staphylococcus, Lactobacillus and Bacteroides*.

The average age of the patients was 55.5 (IQR (interquartile range) 51.25 – 60.00). Among them, men - 72 (56.3%), women - 56 (43.4%) (**Table 1**; **Figure 4**). The results of our study indicate that patients infected with the Omicron variant of the virus (period of Omicron variant predominance in our region) have a higher abundance of certain gut bacteria genera compared to those infected with the Delta variant. Specifically, we found that the Omicron group had a significantly higher abundance of the genus *Bifidobacterium* (P=0.000), *Bacteroides* (P=0.023), *Klebsiella* (P=0.000), *Enterobacter* (P=0.016), and *Lactobacillus* (P=0.005). In contrast, patients in the Delta group had a significantly higher abundance of the genus *Clostridium* (P=0.000), *Enterococcus* (P=0.000), and *Candida* (P=0.003) compared to the Omicron group (**Figure 5**).

**Table 1:** Basic characteristics of the study population.

Group	Years		Female, n (%)	Male, n (%)	All, n
	Median	IQR			
Delta variant	56	51.75 – 60.00	17 (40.4%)	25 (59.6%)	42
Omicron variant	55	51.00 – 60.25	39 (45.3%)	47 (54.7%)	86
Antibiotic-Treated group	56	52.00 – 60.25	25 (43.1%)	33 (56.9%)	58
Non-Antibiotic-Treated group	55	51.25 – 59.75	31 (44.2%)	39 (55.8%)	70
COVID-19 with T2D	56	49.5 – 60.00	24 (53.3%)	21 (46.7%)	45
COVID-19 without T2D	55	52.00 – 60.00	32 (38.5%)	51 (61.5%)	83
Metformin-treated patients with T2D and COVID-19 with antibiotic treatment	56	49.00 – 60.25	11 (50%)	11 (50%)	22
Metformin-treated patients with T2D and COVID-19 without antibiotic treatment	58	51.00 – 60.75	7 (58.3%)	5 (41.6%)	12
Total	55.5	51.25 – 60.00	56 (43.8%)	72 (56.3%)	128

**Figure 6:**
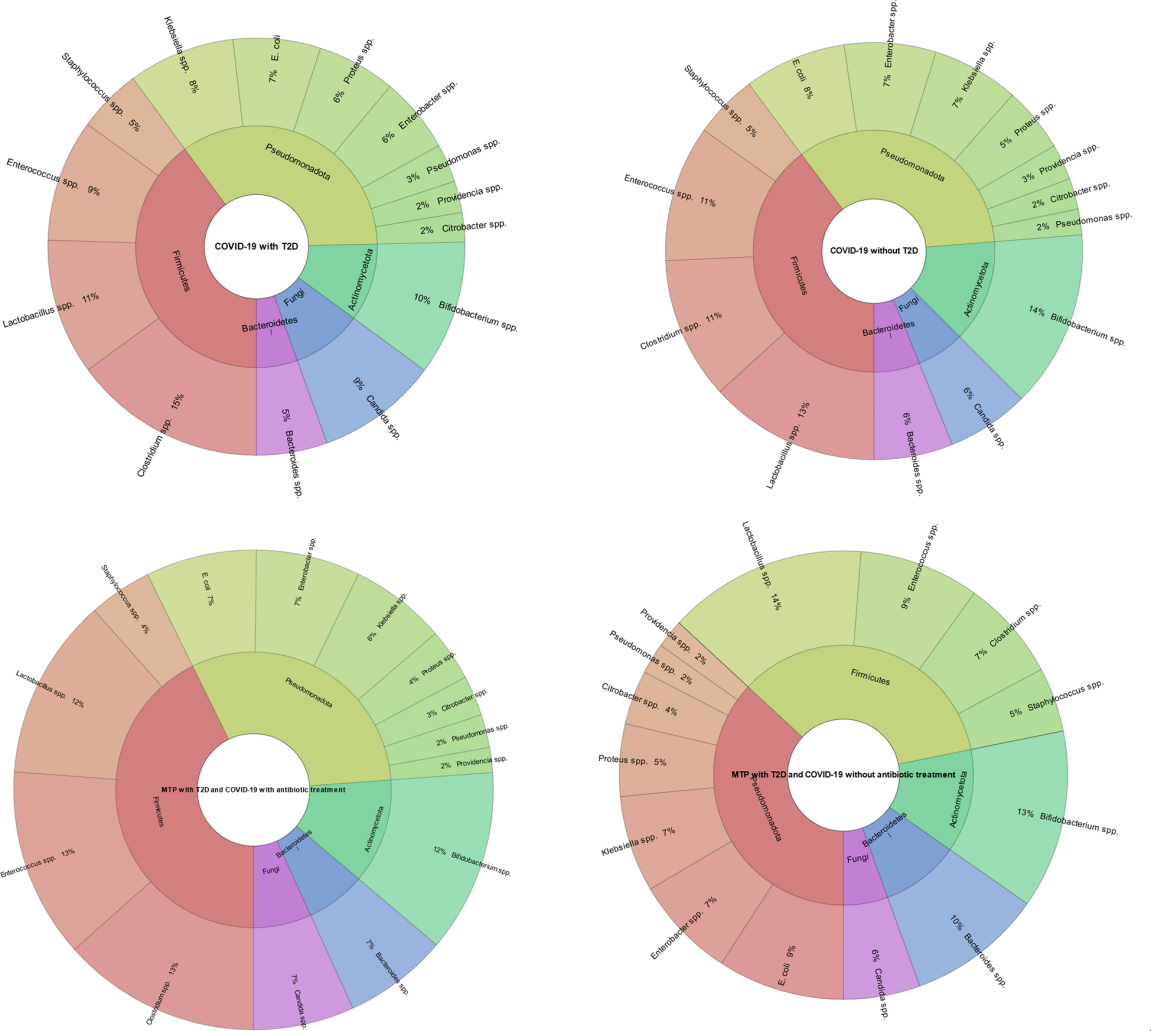
Comparison of gut microbiota composition in patients with COVID-19 and T2D, and gut microbiota composition in metformin-treated patients. The results indicate that the patients with COVID-19 and T2D had a lower abundance of the genus *Bifidobacterium* (P=0.000) and *Lactobacillus* (P=0.000) and a higher abundance of the genus *Candida* (P=0.000) compared to patients with COVID-19 but no T2D. Also, the metformin-treated group without antibiotic treatment had a higher abundance of the genus *Bacteroides* (P=0.000), *E. coli* (P=0.005), *Lactobacillus* (P=0.000), and a lower abundance of the genus *Clostridium* (P=0.000) and *Enterococcus* (P=0.000) compared to the metformin-treated group with antibiotic treatment. This figure illustrates the impact of metformin treatment and antibiotic treatment on gut microbiome composition in patients with COVID-19 and T2D and the difference in gut microbiome composition between patients with COVID-19 and T2D and patients with COVID-19 but no T2D.

Our study’s findings suggest that patients treated with antibiotics have a different gut microbiome composition compared to those who were not treated with antibiotics. Specifically, we found that the antibiotic-treated group had a higher abundance of the genus *Enterococcus* (P=0.000), Candida (P=0.003) and *Clostridium* (P=0.000). In contrast, patients in the non-antibiotic-treated group had a higher abundance of the genus *Proteus* (P=0.000), *Klebsiella* (P=0.000), *Citrobacter* (P=0.028), *Staphylococcus* (P=0.000), *Lactobacillus* (P=0.000) and *Bacteroides* (P=0.000) compared to the antibiotic-treated group. Also, we found that patients with COVID-19 and T2D had a lower abundance of the genus *Bifidobacterium* (P=0.000) and *Lactobacillus* (P=0.000) and a higher abundance of the genus *Candida* (P=0.000) compared to patients with COVID-19 but no T2D (**Figure 6**).

Our study found that metformin-treated patients with T2D and COVID-19 without antibiotic treatment had a different gut microbiome composition compared to metformin-treated patients with T2D and COVID-19 who also received antibiotic treatment. Specifically, we found that the metformin-treated group without antibiotic treatment had a higher abundance of the genus *Bacteroides* (P=0.000), E. coli (P=0.005), *Lactobacillus* (P=0.000), and a lower abundance of the genus *Clostridium* (P=0.000) and *Enterococcus* (P=0.000) compared to the metformin-treated group with antibiotic treatment (**Figure 6**).

**Figure 7:**
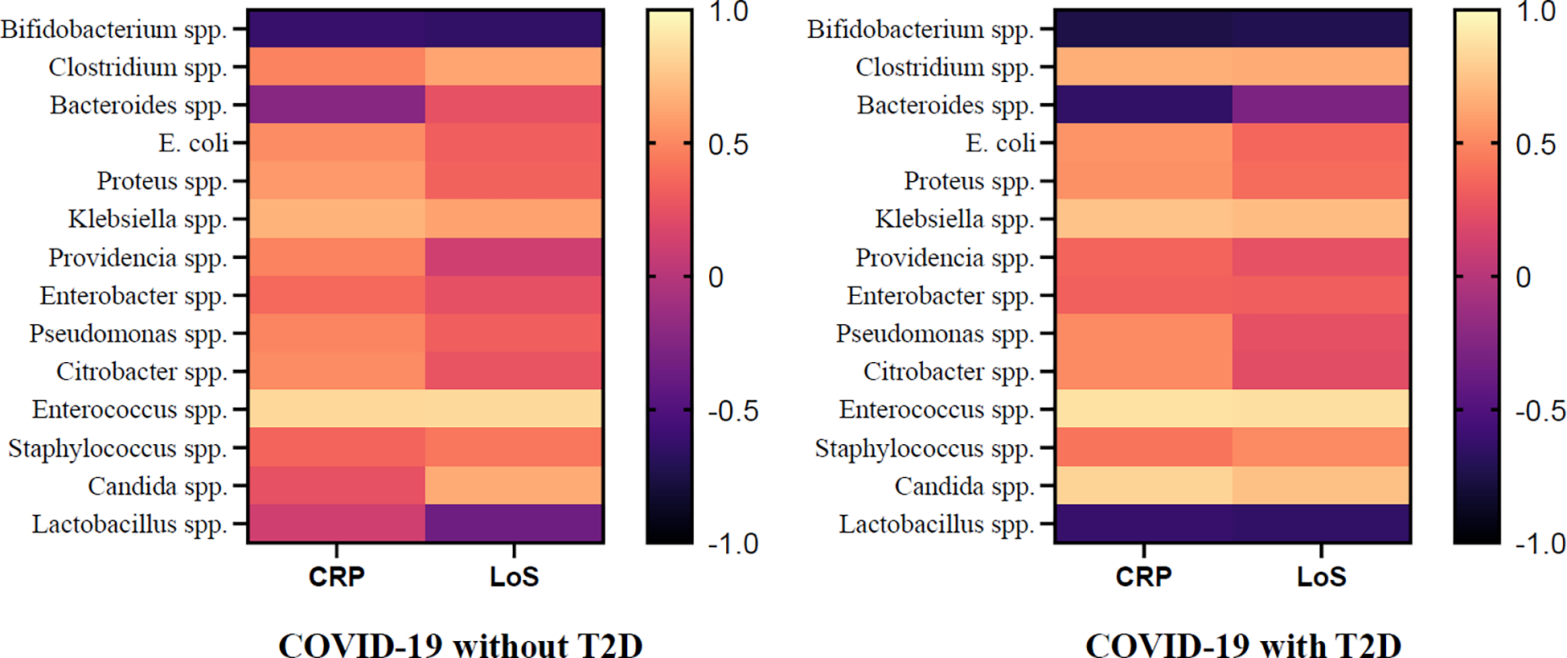
Association of CRP, LoS and Abundance of Gut Microorganisms in COVID-19 Patients with and without T2D.

Additionally, the results revealed a positive correlation between the abundance of the genus *Klebsiella* spp. (r= 0.68, p = 0.002) and *Enterococcus* spp. (r= 0.84, p=0.005) and CRP levels in COVID-19 patients without T2D, while the abundance of *Bifidobacterium* spp. was found to have a negative correlation with CRP levels (r=-0.62, p=0.032).Similarly, the abundance of *Clostridium* spp. (r= 0.62, p=0.022), *Klebsiella* spp. (r= 0.61, p=0.024), *Enterococcus* spp. (r= 0.85, p=0.004), and *Candida* spp. (r= 0.65, p=0.043) were positively correlated with the LoS, while the abundance of *Bifidobacterium* spp. was negatively correlated with LoS (r= -0.65, p = 0.045). In addition to the findings in COVID-19 patients without T2D, the study also investigated the correlation between gut microbiota and CRP and LoS in COVID-19 patients with T2D. The results showed that in this patient population, the abundance of genus *Clostridium* spp. (r=0.66, p = 0.038), *Klebsiella* spp. (r=0.75, p= 0.003), *Enterococcus* spp. (r= 0.88, p < 0.001), *Candida* spp. (r=0.82, p= 0.002) was positively correlated with CRP levels, while the abundance of genus *Bifidobacterium* spp. (r= - 0.75, p = 0.028) and *Lactobacillus* spp. (r= -0.62, p= 0.032) was negatively correlated with CRP. Similarly, the abundance of *Klebsiella* spp. (r=0.72, p=0.022), *Enterococcus* spp. (r=0.87, p < 0.001) and *Candida* spp. (r=0.74, p=0.005) was positively correlated with LoS, and *Bifidobacterium* spp. (r= - 0.72, p=0.007) was negatively correlated with LoS (**Figure 7**).

## 4 Discussion

In this article, we showed changes in the gut microbiota in patients with COVID-19, using the culture-based method. Culture-based gut microbiome research is a method of studying the gut microbiome by isolating and culturing bacteria from fecal samples. This method can provide information on the growth and metabolic characteristics of specific bacteria, which can be useful for identifying and characterizing new bacterial species or strains. However, it also has some limitations, particularly when compared to PCR and 16S rRNA sequencing (Ito et al., 2019). One major limitation of culture-based gut microbiome research is that it is limited to only a small subset of the total gut microbiome. This is because many gut bacteria are difficult or impossible to culture in laboratory conditions, and so are not captured by this method. On the other hand, PCR and 16S rRNA sequencing can provide a more comprehensive view of the gut microbiome by amplifying and sequencing the 16S rRNA gene, which is present in all bacteria (Hitch et al., 2021).

An important finding that should be noted in our research is the significant increase *Enterococcus* spp. in patients with the Delta variant. Recent studies have suggested a possible association between *Enterococcus* and COVID-19. Some studies have found that patients with COVID-19 have higher levels of *Enterococcus* in their gut microbiome compared to healthy controls (Toc et al., 2022). Other studies have found that certain strains of Enterococcus are more common in COVID-19 patients than in healthy individuals (Giacobbe et al., 2021). The exact role of *Enterococcus* in COVID-19 is not yet fully understood, but some researchers believe that it may play a role in the development of severe illness. For example, it has been proposed that Enterococcus may contribute to the development of cytokine storm, which is a severe immune response that can occur in some COVID-19 patients (Zuo et al., 2020; Gago et al., 2022; Righi et al., 2022; Toc et al., 2022). The exact mechanism by which *Enterococcus* may contribute to cytokine storm is not well understood, but it is suggested that it may involve the activation of Toll-like receptors (TLRs), which are proteins on the surface of immune cells that recognize specific microbial products (Guiton et al., 2013). It’s also suggested that *Enterococcus* may increase the production of pro-inflammatory cytokines such as TNF-alpha and IL-6, which have been shown to be elevated in patients with COVID-19 and are associated with severe disease (Strickertsson et al., 2013; Sparo et al., 2014). In our opinion, the possible increase in the abundance of *Enterococcus* spp. is related to the widespread use of carbapenems during the delta wave of COVID-19 in our region (Righi et al., 2022).

*Klebsiella pneumoniae*, a member of the *Enterobacteriaceae* family, is considered an opportunistic pathogen and has been frequently identified in high levels in the gut of critically ill COVID-19 patients (Tang et al., 2020). An overgrowth of pathobionts such as this can weaken the gut barrier, increasing the risk of bloodstream infections in these patients (Langford et al., 2020). Additionally, a number of studies have reported co-infections involving *Klebsiella* spp. and *Enterococcus* spp. in a significant proportion of COVID-19 patients. It is worth noting that in critical cases, these co-infections have been linked to up to 50% of deaths (Zhou et al., 2020).

Also, to some extent, the use of antibiotics is associated with an increase in the abundance of *Clostridium* spp. There are some studies in which Hospitalized patients with COVID-19 showed an increased abundance of *Clostridium ramosum* compared with those hospitalized with other types of viral pneumonia (Zuo et al., 2020). *Clostridium ramosum* can induce RORγt expression in Foxp3^+^ Treg cells. RORγt^+^ Foxp3+ Treg cells downregulate T_H_1-, T_H_2-, and T_H_17 cell-type immune responses (Ohnmacht et al., 2015; Sefik et al., 2015). *Clostridium butyricum, Clostridium leptum* as a butyrate-producing bacterium were decreased significantly in COVID-19 patients in some studies (Tang et al., 2020). Butyrate is one of the short-chain fatty acids (SCFAs) which can activate anti-inflammatory responses of immune cells, inhibit inflammatory signalling pathways and maintain the integrity of the gut barrier to prevent translocation of gut endotoxins and bacteria into the circulation, thereby alleviating local and systemic inflammatory responses (Geirnaert et al., 2017). Empiric antibiotic treatments for microbial infections in hospitalized patients with COVID-19 in addition to experimental antiviral and immunomodulatory drugs may increase Clostridioides difficile infection (CDI) (Azimirad et al., 2021).

Previous research found that the gut microbiota was moderately dysregulated in Chinese and Danish T2D patients. Specifically, Chinese patients showed an increase in multiple pathogenic bacteria, such as *Clostridium hathewayi, Clostridium symbiosum* and *Escherichia coli*, while healthy controls had a high abundance of butyrate-producing bacteria (Qin et al., 2012). Compared with individuals with normal glucose regulation, the most significant feature of the gut microbiota in Danish patients with prediabetes was the decreased abundance of *Clostridium* genus and *A. muciniphila* (Allin et al., 2018). In our study, patients with COVID-19 and T2D had significantly higher abundance of *Clostridium* spp. than patients without diabetes.

Several studies have shown that metformin treatment can change the structure of the gut microbiome (Kamyshnyi et al., 2021). In particular, some studies have shown that the concentration of SCFAs, such as propionate, is increased in people who take metformin compared to those who do not (Zhernakova et al., 2016). Another study found that after four months of treatment with metformin, the levels of fecal butyrate and propionate were higher in men who received metformin compared to those who received a placebo, which suggests that metformin can affect the levels of fermentative metabolites involved in regulating human metabolism (Wu et al., 2017). Additionally, research has shown that in patients with T2D who were given metformin or a placebo for four months, there was an increase in the abundance of certain types of bacteria such as *Escherichia* spp. and *Bilophila wadsworthia*, along with a decrease in other types such as *Intestinibacter* spp. and *Clostridium* spp (Forslund et al., 2015; Wu et al., 2017).. Similar changes in the gut microbiota were observed in our study.

Notably, some specific *Bacteroides* spp., capable of down-regulating ACE2 expression in the murine gut, are inversely correlated with the SARS-CoV-2 load (Zuo et al., 2020). Also, *Bacteroides fragilis*, promotes Treg induction and IL-10-mediated anti-inflammatory responses from T cells and DCs in a TLR2-dependent manner (Round and Mazmanian, 2010; Round et al., 2011). Based on these findings, it is suggested that Bacteroides species have an anti-inflammatory effect and play a role in managing inflammation through the gut-lung axis.

Gastrointestinal symptoms, such as diarrhea, which occur for an extended period in people with COVID-19, have been linked to a decrease in the variety and abundance of gut bacteria, immune system imbalances, and delayed clearance of the SARS-CoV-2 virus (Gu et al., 2020; Lamers et al., 2020). The relationship between the gut and respiratory systems, known as the gut-lung axis, is thought to play a role in the body’s immune response to the virus (Budden et al., 2017). Studies have shown that an imbalance in gut bacteria, called dysbiosis, can lead to higher mortality rates in other respiratory infections, as it can exacerbate inflammation and weaken the lung’s and gut’s ability to regulate and reduce inflammation (Grayson et al., 2018). Therefore, given that both the respiratory and gastrointestinal tracts can be affected by the virus and that dysbiosis and inflammation can occur, it is reasonable to consider additional therapies that focus on modulating the gut microbiome, such as using metformin, probiotics, or fecal microbial transplantation to re-establish a healthy balance of gut bacteria, as a potential treatment option (Baindara et al., 2021).

Additionally, the idea that probiotics, which have been studied and recommended for respiratory tract infections, could potentially have a beneficial effect against COVID-19 is being explored. Studies have shown that an imbalance in gut bacteria, known as dysbiosis, can worsen lung pathology and lead to secondary infections during influenza virus infections (Sencio et al., 2020). Additionally, some COVID-19 patients have reported an imbalance in gut bacteria, including a decrease in natural probiotic species such as *Lactobacillus* and *Bifidobacterium* (Xu et al., 2020).

## 5 Limitations

This study has several limitations that should be taken into consideration when interpreting the results. Firstly, the sample size of 128 COVID-19 patients with T2D is relatively small and may not be representative of the general population. Additionally, the study only used a culture-based method to analyze the gut microbiota changes, which may not capture all the bacterial taxa present in the gut. Furthermore, the study only looked at the correlation between gut microbiota, CRP levels, and length of stay and did not establish causality. Finally, this study is a cross-sectional study, so it’s unable to establish temporal relationships. Future studies with larger sample sizes and using more advanced techniques, such as 16S rRNA sequencing, are needed to confirm these findings and investigate the mechanisms underlying the observed associations.

## Data availability statement

The raw data supporting the conclusions of this article will be made available by the authors, without undue reservation.

## Ethics statement

The studies involving human participants were reviewed and approved by Transcarpathian Regional Clinical Infectious Hospital Ethics Committee. The patients/participants provided their written informed consent to participate in this study.

## Author contributions

PP, AK contributed the conceptualization and the original idea of this manuscript. PP, AK, and NK contributed the methodology and reviewed the literature. PP, AK, and NK were involved in validation and revised and validated the literature findings. PP and AK performed the formal analysis. PP and AK conducted the investigation. PP, AK, and NK were involved in data curation, writing—original draft preparation, review and editing, and supervision. PP and AK performed the visualization and contributed to project administration. All authors contributed to the article and approved the submitted version.

## References

[B6] Al-Kuraishy H. M. Al-Gareeb A. I. Alblihed M. Guerreiro S. G. Cruz-Martins N. Batiha G. E.-S. (2021). COVID-19 in relation to hyperglycemia and diabetes mellitus. J. Front. Cardiovasc. Med. 8, 644095. doi: 10.3389/fcvm.2021.644095 PMC8189260 34124187

[B32] Allin K. H. Tremaroli V. Caesar R. Jensen B. A. Damgaard M. T. Bahl M. I.. (2018). Aberrant intestinal microbiota in individuals with prediabetes. J. Diabetologia. 61 (4), 810–820. doi: 10.1007/s00125-018-4550-1 29379988 PMC6448993

[B30] Azimirad M. Noori M. Raeisi H. Yadegar A. Shahrokh S. Asadzadeh Aghdaei H.. (2021). How does COVID-19 pandemic impact on incidence of clostridioides difficile infection and exacerbation of its gastrointestinal symptoms? J. Front. Med., 8, 2569. doi: 10.3389/fmed.2021.775063 PMC8710593 34966759

[B43] Baindara P. Chakraborty R. Holliday Z. M. Mandal S. M. Schrum A. G. (2021). Oral probiotics in coronavirus disease 2019: Connecting the gut-lung axis to viral pathogenesis, inflammation, secondary infection and clinical trials. New Microbes New infections. 40, 100837. doi: 10.1016/j.nmni.2021.100837 33425362 PMC7785423

[B9] Belkaid Y. Hand T. W. (2014). Role of the microbiota in immunity and inflammation. J. Cell Metab. 157 (1), 121–141. doi: 10.1016/j.cell.2014.03.011 PMC4056765 24679531

[B41] Budden K. F. Gellatly S. L. Wood D. L. Cooper M. A. Morrison M. Hugenholtz P.. (2017). Emerging pathogenic links between microbiota and the gut–lung axis. J. Nat. Rev. Microbiol. 15 (1), 55–63. doi: 10.1038/nrmicro.2016.142 27694885

[B4] Corrao S. Pinelli K. Vacca M. Raspanti M. Argano C. (2021). Type 2 diabetes mellitus and COVID-19: A narrative review. Front. Endocrinol. (Lausanne). 12, 609470. doi: 10.3389/fendo.2021.609470 33868163 PMC8044543

[B11] Cruz C. S. Ricci M. F. Vieira A. T. (2021). Gut microbiota modulation as a potential target for the treatment of lung infections. Front. Pharmacol. 12, 724033. doi: 10.3389/fphar.2021.724033 34557097 PMC8453009

[B500] Dunn J. J. (2016). Guidelines for biochemical identification of aerobic bacteria. Clin. Microbiol. Procedures Handb. 3.16.1–3.13.5. doi: 10.1128/9781683670438

[B36] Forslund K. Hildebrand F. Nielsen T. Falony G. Le Chatelier E. Sunagawa S.. (2015). Disentangling type 2 diabetes and metformin treatment signatures in the human gut microbiota. Nature. 528 (7581), 262–266. doi: 10.1038/nature15766 26633628 PMC4681099

[B19] Gago J. Filardo T. D. Conderino S. Magaziner S. J. Dubrovskaya Y. Inglima K.. (2022). Pathogen species is associated with mortality in nosocomial bloodstream infection in patients with COVID-19. Open Forum Infect. Dis. 9 (6), ofac083. doi: 10.1093/ofid/ofac083 35607701 PMC8992347

[B29] Geirnaert A. Calatayud M. Grootaert C. Laukens D. Devriese S. Smagghe G.. (2017). Butyrate-producing bacteria supplemented *in vitro* to crohn’s disease patient microbiota increased butyrate production and enhanced intestinal epithelial barrier integrity. J. Sci. Rep. 7 (1), 1–14. doi: 10.1038/s41598-017-11734-8 PMC5597586 28904372

[B17] Giacobbe D. R. Labate L. Tutino S. Baldi F. Russo C. Robba C.. (2021). Enterococcal bloodstream infections in critically ill patients with COVID-19: A case series. Ann. Med. 53 (1), 1779–1786. doi: 10.1080/07853890.2021.1988695 34637370 PMC8519517

[B42] Grayson M. H. Camarda L. E. Hussain S.-R. A. Zemple S. J. Hayward M. Lam V.. (2018). Intestinal microbiota disruption reduces regulatory T cells and increases respiratory viral infection mortality through increased IFNγ production. J. Front. Immunol. 9, 1587. doi: 10.3389/fimmu.2018.01587 PMC6048222 30042764

[B501] Gu S. Chen Y. Wu Z. Chen Y. Gao H. Lv L.. (2020). Alterations of the gut microbiota in patients with coronavirus disease 2019 or H1N1 influenza. Clin. Infect. Dis. 71(10), 2669–2678. doi: 10.1093/cid/ciaa709 32497191 PMC7314193

[B21] Guiton P. S. Hannan T. J. Ford B. Caparon M. G. Hultgren S. J. (2013). Enterococcus faecalis overcomes foreign body-mediated inflammation to establish urinary tract infections. Infection immunity. 81 (1), 329–339. doi: 10.1128/IAI.00856-12 23132492 PMC3536162

[B15] Hitch T. C. A. Afrizal A. Riedel T. Kioukis A. Haller D. Lagkouvardos I.. (2021). Recent advances in culture-based gut microbiome research. Int. J. Med. Microbiol. 311 (3), 151485. doi: 10.1016/j.ijmm.2021.151485 33689954

[B2] Hu B. Guo H. Zhou P. Shi Z.-L. (2021). Characteristics of SARS-CoV-2 and COVID-19. J. Nat. Rev. Microbiol. 19 (3), 141–154. doi: 10.1038/s41579-020-00459-7 PMC7537588 33024307

[B12] Iatcu C. O. Steen A. Covasa M. (2021). Gut microbiota and complications of type-2 diabetes. Nutrients. 14 (1). doi: 10.3390/nu14010166 PMC8747253 35011044

[B14] Ito T. Sekizuka T. Kishi N. Yamashita A. Kuroda M. (2019). Conventional culture methods with commercially available media unveil the presence of novel culturable bacteria. Gut Microbes 10 (1), 77–91. doi: 10.1080/19490976.2018.1491265 30118379 PMC6363062

[B33] Kamyshnyi O. Matskevych V. Lenchuk T. Strilbytska O. Storey K. Lushchak O. (2021). Metformin to decrease COVID-19 severity and mortality: Molecular mechanisms and therapeutic potential. Biomedicine Pharmacotherapy. 144, 112230. doi: 10.1016/j.biopha.2021.112230 34628168 PMC8492612

[B39] Lamers M. M. Beumer J. van der Vaart J. Knoops K. Puschhof J. Breugem T. I.. (2020). SARS-CoV-2 productively infects human gut enterocytes. Science. 369 (6499), 50–54. doi: 10.1126/science.abc1669 32358202 PMC7199907

[B25] Langford B. J. So M. Raybardhan S. Leung V. Westwood D. MacFadden D. R.. (2020). Bacterial co-infection and secondary infection in patients with COVID-19: A living rapid review and meta-analysis. J. Clin. Microbiol. infection 26 (12), 1622–1629. doi: 10.1016/j.cmi.2020.07.016 PMC7832079 32711058

[B5] Lima-Martínez M. M. Carrera Boada C. Madera-Silva M. D. Marín W. Contreras M. (2021). COVID-19 and diabetes: A bidirectional relationship. Clinica e investigacion en arteriosclerosis: publicacion oficial la Sociedad Espanola Arteriosclerosis. 33 (3), 151–157. doi: 10.1016/j.arteri.2020.10.001 PMC7598432 33303218

[B10] Mousa W. K. Chehadeh F. Husband S. (2022). Microbial dysbiosis in the gut drives systemic autoimmune diseases. Front. Immunol. 13, 906258. doi: 10.3389/fimmu.2022.906258 36341463 PMC9632986

[B28] Ohnmacht C. Park J.-H. Cording S. Wing J. B. Atarashi K. Obata Y.. (2015). The microbiota regulates type 2 immunity through RORγt+ T cells. Science. 349 (6251), 989–993. doi: 1010.1126/science.aac4263 26160380 10.1126/science.aac4263

[B7] Petakh P. Griga V. Mohammed I. B. Loshak K. Poliak I. Kamyshnyiy A. (2022). Effects of metformin, insulin on hematological parameters of COVID-19 patients with type 2 diabetes. Med. Arch. (Sarajevo Bosnia Herzegovina). 76 (5), 329–332. doi: 10.5455/medarh.2022.76.329-332 PMC9760238 36545453

[B8] Petakh P. Kamyshna I. Nykyforuk A. Yao R. Imbery J. F. Oksenych V.. (2022). Immunoregulatory intestinal microbiota and COVID-19 in patients with type two diabetes: A double-edged sword. J. Viruses. 14 (3), 477. doi: 10.3390/v14030477 PMC8955861 35336884

[B31] Qin J. Li Y. Cai Z. Li S. Zhu J. Zhang F.. (2012). A metagenome-wide association study of gut microbiota in type 2 diabetes. Nature. 490 (7418), 55–60. doi: 10.1038/nature11450 23023125

[B20] Righi E. Lambertenghi L. Gorska A. Sciammarella C. Ivaldi F. Mirandola M.. (2022). Impact of COVID-19 and antibiotic treatments on gut microbiome: A role for enterococcus spp. J. Biomedicines. 10 (11), 2786. doi: 10.3390/biomedicines10112786 PMC9687172 36359311

[B37] Round J. L. Lee S. M. Li J. Tran G. Jabri B. Chatila T. A.. (2011). The toll-like receptor 2 pathway establishes colonization by a commensal of the human microbiota. J. Science. 332 (6032), 974–977. doi: 10.1073/pnas.0909122107 PMC3164325 21512004

[B38] Round J. L. Mazmanian S. K. (2010). Inducible Foxp3+ regulatory T-cell development by a commensal bacterium of the intestinal microbiota. Proc. Natl. Acad. Sci. 107 (27), 12204–12209. doi: 10.1073/pnas.0909122107 20566854 PMC2901479

[B27] Sefik E. Geva-Zatorsky N. Oh S. Konnikova L. Zemmour D. McGuire A. M.. (2015). Individual intestinal symbionts induce a distinct population of RORγ+ regulatory T cells. Science. 349 (6251), 993–997. doi: 10.1126/science.aaa9420 26272906 PMC4700932

[B44] Sencio V. Barthelemy A. Tavares L. P. Machado M. G. Soulard D. Cuinat C.. (2020). Gut dysbiosis during influenza contributes to pulmonary pneumococcal superinfection through altered short-chain fatty acid production. Cell Rep. 30 (9), 2934–47.e6. doi: 10.1016/j.celrep.2020.02.013 32130898

[B1] She J. Jiang J. Ye L. Hu L. Bai C. Song Y. (2020). 2019 novel coronavirus of pneumonia in wuhan, China: Emerging attack and management strategies. J. Clin. Trans. Med. 9 (1), 1–7. doi: 10.1186/s40169-020-00271-z PMC7033263 32078069

[B3] Shrestha L. B. Foster C. Rawlinson W. Tedla N. Bull R. A. (2022). Evolution of the SARS-CoV-2 omicron variants BA. 1 to BA. 5: Implications for immune escape and transmission. J. Rev. Med. Virology. 32 (5), e2381. doi: 10.1002/rmv.2381 PMC9349777 35856385

[B23] Sparo M. Delpech G. Batisttelli S. Basualdo J. (2014). Immunomodulatory properties of cell wall extract from enterococcus faecalis CECT7121. Braz. J. Infect. diseases: an Off. Publ. Braz. Soc. Infect. Diseases. 18 (5), 551–555. doi: 10.1016/j.bjid.2014.05.005 PMC9428229 24907474

[B22] Strickertsson J. A. B. Desler C. Martin-Bertelsen T. Machado A. M. D. Wadstrøm T. Winther O.. (2013). Enterococcus faecalis infection causes inflammation, intracellular oxphos-independent ROS production, and DNA damage in human gastric cancer cells. PloS One 8 (4), e63147. doi: 10.1371/journal.pone.0063147 23646188 PMC3639970

[B24] Tang L. Gu S. Gong Y. Li B. Lu H. Li Q.. (2020). Clinical significance of the correlation between changes in the major intestinal bacteria species and COVID-19 severity. Engineering. 6 (10), 1178–1184. doi: 10.1016/j.eng.2020.05.013 33520333 PMC7832131

[B16] Toc D. A. Mihaila R. M. Botan A. Bobohalma C. N. Risteiu G. A. Simut-Cacuci B. N.. (2022). Enterococcus and COVID-19: The emergence of a perfect storm? J. Int. J. Trans. Med. 2 (2), 220–229. doi: 10.3390/ijtm2020020

[B35] Wu H. Esteve E. Tremaroli V. Khan M. T. Caesar R. Mannerås-Holm L.. (2017). Metformin alters the gut microbiome of individuals with treatment-naive type 2 diabetes, contributing to the therapeutic effects of the drug. Nat. Med. 23 (7), 850–858. doi: 10.1038/nm.4345 28530702

[B45] Xu K. Cai H. Shen Y. Ni Q. Chen Y. Hu S.. (2020). [Management of COVID-19: the zhejiang experience]. Zhejiang da xue xue bao Yi xue ban = J. Zhejiang Univ. Med. Sci. 49 (2), 147–157. doi: 10.3785/j.issn.1008-9292.2020.02.02 PMC8800711 32391658

[B34] Zhernakova A. Kurilshikov A. Bonder M. J. Tigchelaar E. F. Schirmer M. Vatanen T.. (2016). Population-based metagenomics analysis reveals markers for gut microbiome composition and diversity. J. Science. 352 (6285), 565–569. doi: 10.1126/science.aad3369 27126040 PMC5240844

[B26] Zhou F. Yu T. Du R. Fan G. Liu Y. Liu Z.. (2020). Clinical course and risk factors for mortality of adult inpatients with COVID-19 in wuhan, China: A retrospective cohort study. J. Lancet Diabetes endocrinology. 395 (10229), 1054–1062. doi: 10.1016/S0140-6736(20)30566-3 PMC7270627 32171076

[B18] Zuo T. Zhang F. Lui G. C. Yeoh Y. K. Li A. Y. Zhan H.. (2020). Alterations in gut microbiota of patients with COVID-19 during time of hospitalization. Gastroenterology. 159 (3), 944–55. e8. doi: 10.1053/j.gastro.2020.05.048 32442562 PMC7237927

