# Supplementary material for: Gut microbiota in patients with COVID-19 and type 2 diabetes: A culture-based method
Source: Front Cell Infect Microbiol. 2023 Feb 9;13:1142578. doi: 10.3389/fcimb.2023.1142578 (PMC9947359; doi:10.3389/fcimb.2023.1142578)

Supplementary Material

**Gut Microbiota in Patients with COVID-19 and Type 2 Diabetes: A Culture-Based Method**

**Pavlo Petakh^1,2*^, Nazarii Kobyliak^3,4^, Aleksandr Kamyshnyi^2*^**

^1^Department of Biochemistry and Pharmacology, Uzhhorod National University, Uzhhorod, Ukraine;

^2^Department of Microbiology, Virology, and Immunology, I. Horbachevsky Ternopil National Medical University, Ternopil, Ukraine;

^3^Medical Laboratory CSD, Kyiv, Ukraine

^4^Endocrinology Department, Bogomolets National Medical University, Kyiv, Ukraine

*** Correspondence:** [pavlo.petakh@uzhnu.edu.ua](mailto:pavlo.petakh@uzhnu.edu.ua) (P.P); [kamyshnyi_om@tdmu.edu.ua](mailto:kamyshnyi_om@tdmu.edu.ua) (A.K)

## Supplementary Table 1. List of nutrient media and reagents.

| **Nutrient Media, Reagents** | | **Manufacturers** |
| --- | --- | --- |
| 1. | Endo agar | Biolife Italiana S.r.l. (Italy) |
| 2. | Mannitol salt agar | Biolife Italiana S.r.l.(Italy) |
| 3. | Bismuth sulfite agar | Pharmaktiv LLC (Ukraine) |
| 4. | Sabouraud dextrose agar with chloramphenicol | Graco Biotech (Poland) |
| 5. | Kligler Iron Agar | HiMedia Laboratories, LLC (India) |
| 6. | Bile Esculin Agar | HiMedia Laboratories, LLC (India |
| 7. | Enterococcus agar | Pharmaktiv LLC (Ukraine) |
| 8. | Iron sulfite agar (Wilson-Blair) | Pharmaktiv LLC (Ukraine) |
| 9. | MRS Agar (Sharpe agar) | HiMedia Laboratories, LLC (India) |
| 10. | Lactobacagar | Pharmaktiv LLC (Ukraine) |
| 11. | Bifidobacterium medium | Pharmaktiv LLC (Ukraine) |
| 12. | Bifidobacterium Selective Count Agar Base | HiMedia Laboratories, LLC (India) |
| 13. | Simmons citrate agar | Pharmaktiv LLC (Ukraine) |
| 14. | Bacteroides Bile Esculin Agar Base | HiMedia Laboratories, LLC (India) |
| 15. | Oxidase test strips | Biolife Italiana S.r.l. (Italy) |
| 16. | Coagulase plasma EDTA | Biolife Italiana S.r.l. (Italy) |
| 17. | Egg yolk emulsion 50% | Biolife Italiana S.r.l. (Italy) |
| 18. | Kovacs’ reagent | Biolife Italiana S.r.l. (Italy) |
| 19. | PYR discs reagent | Key Scientific (US) |
| 20. | Gissa's medium | Pharmaktiv LLC (Ukraine) |
| 21. | Glucose bacteriological | Biolife Italiana S.r.l. (Italy) |
| 22. | Inositol | Biolife Italiana S.r.l. (Italy) |
| 23. | Lactose | Biolife Italiana S.r.l. (Italy) |
| 24. | Maltose | Biolife Italiana S.r.l. (Italy) |
| 25. | Mannitol | Biolife Italiana S.r.l. (Italy) |
| 26. | Sorbitol | Biolife Italiana S.r.l. (Italy) |
| 27. | Sucrose | Biolife Italiana S.r.l. (Italy) |
| 28. | Xylose | Biolife Italiana S.r.l. (Italy) |
| 29. | Amino acid carboxylase broth (with lysine, ornithine, arginine and the control broth without amino acids) | Pharmaktiv LLC (Ukraine) |
| 30. | Novobiocin discs | HiMedia Laboratories, LLC (India) |
| 31. | Buffered 0.85% NaCl solution | Graco Biotech (Poland) |
| 32. | Phenylalanine agar | Pharmaktiv LLC (Ukraine) |
| 33. | Christensen citrate agar | Pharmaktiv LLC (Ukraine) |
| 34. | Barritt Reagent A (for VP test) | HiMedia Laboratories, LLC (India) |
| 35. | Barritt Reagent B (for VP test) | HiMedia Laboratories, LLC (India) |
| 36. | Motility Test Medium | HiMedia Laboratories, LLC (India) |


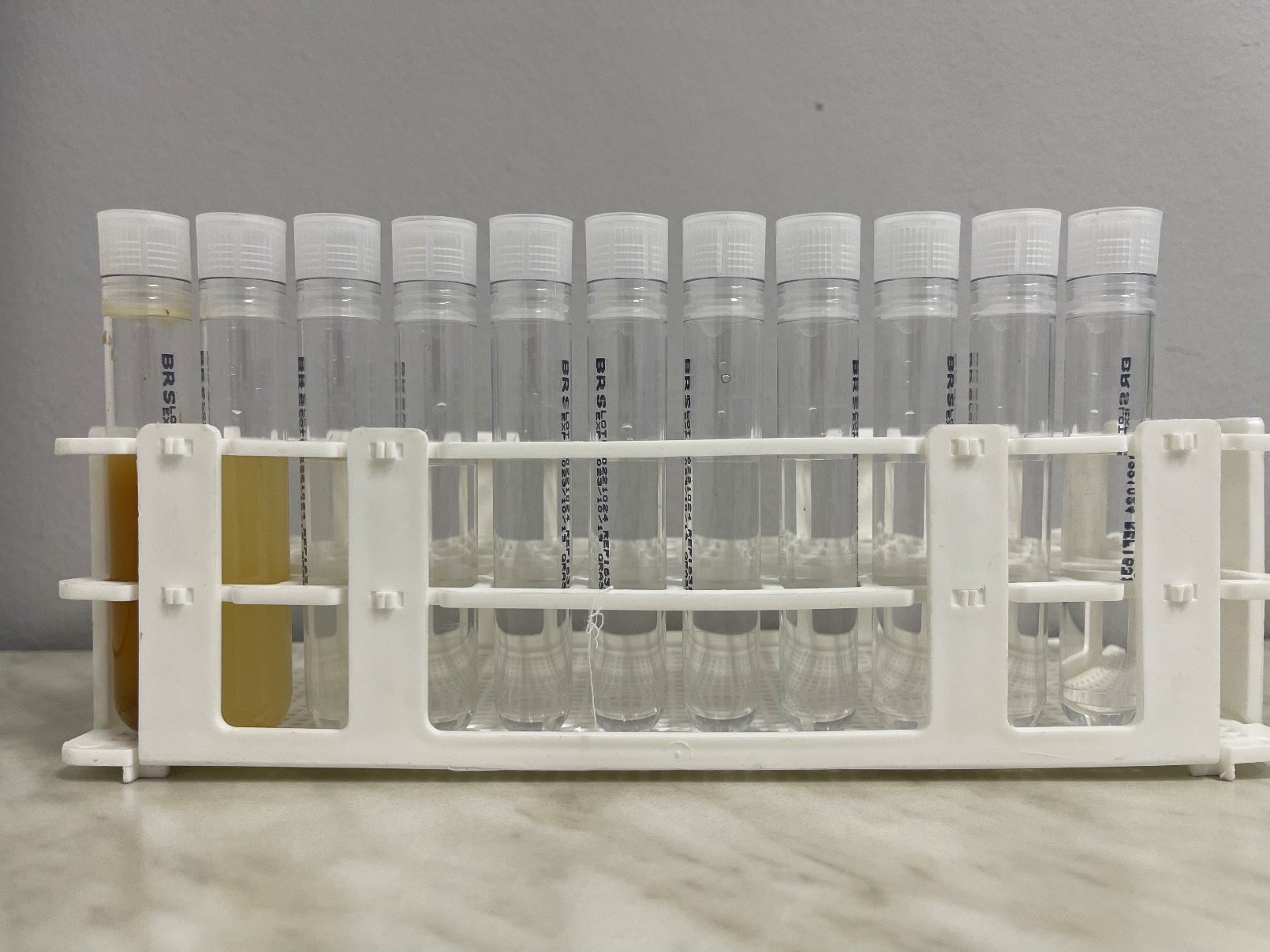
**Supplementary Figure 1. Serial Dilution of Stool Samples.**


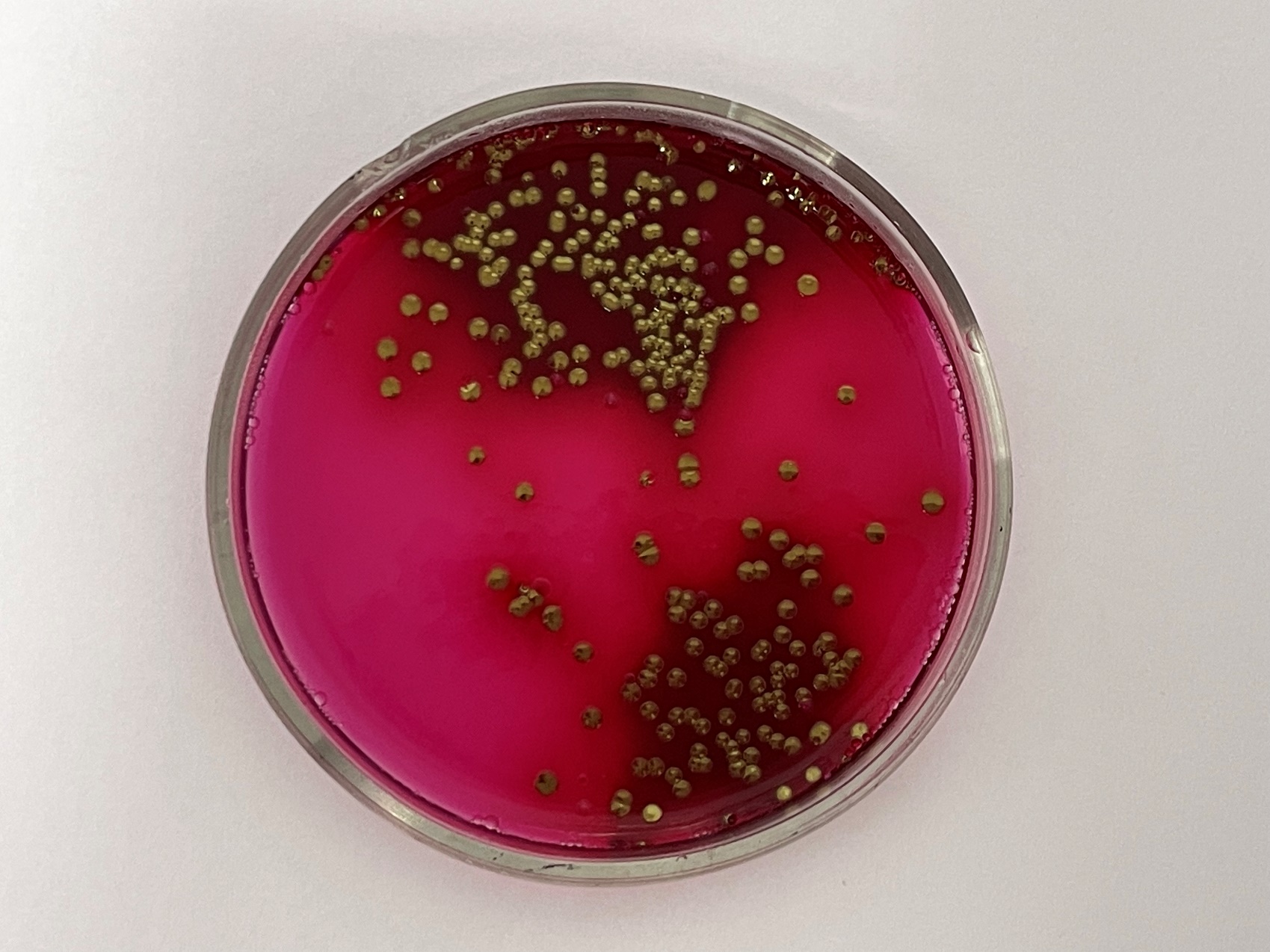
**Supplementary Figure 2. Bacterial Colonies Cultured on Endo Agar (Biolife Italiana S.r.l., Italy).**

**Supplementary Figure 3. Bacterial Colonies Cultured on Endo Agar (Biolife Italiana S.r.l., Italy).**

**
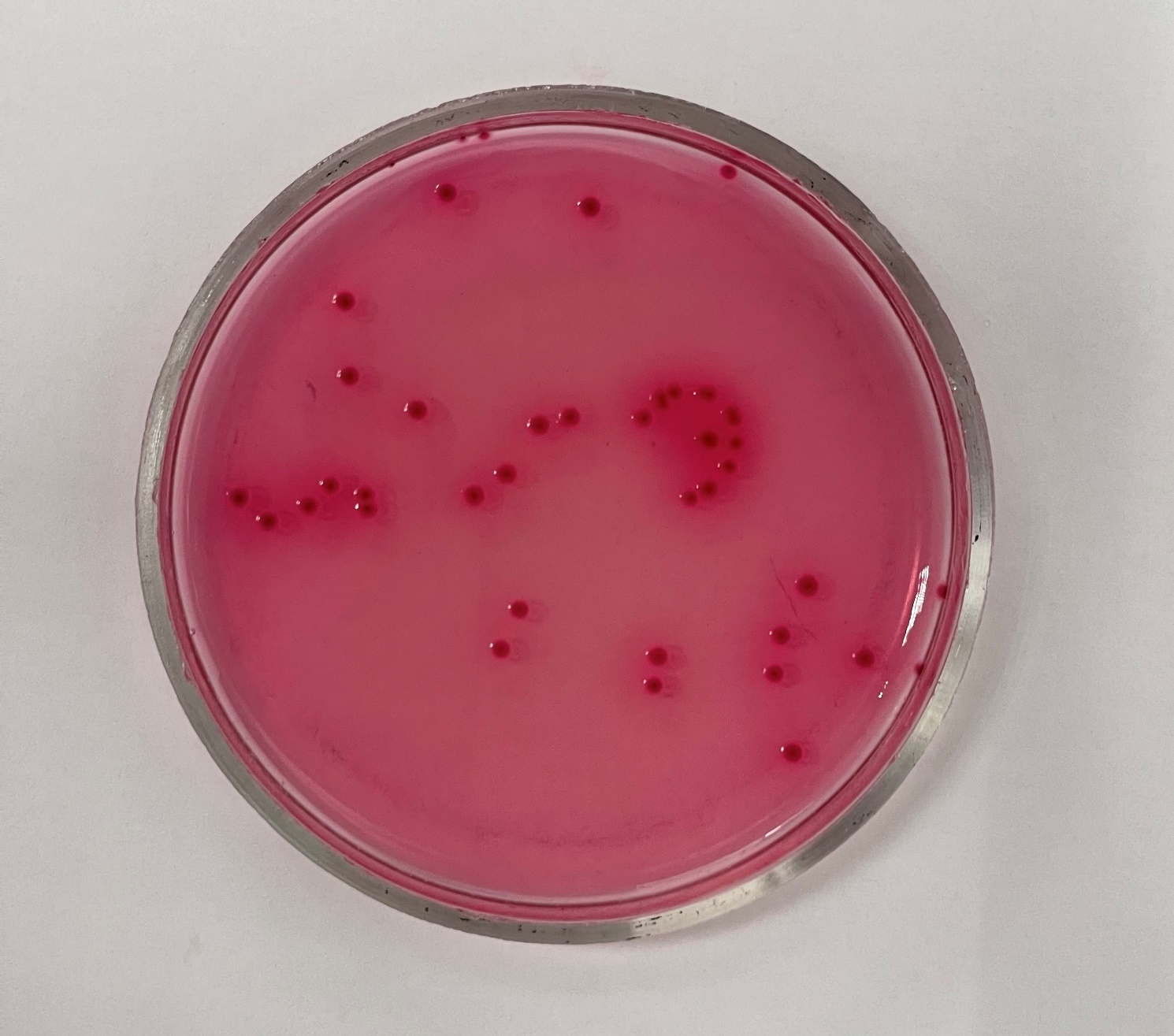
**

**Supplementary Figure 4. Bacterial Colonies Cultured on Enterococcus agar (Pharmaktiv LLC, Ukraine).
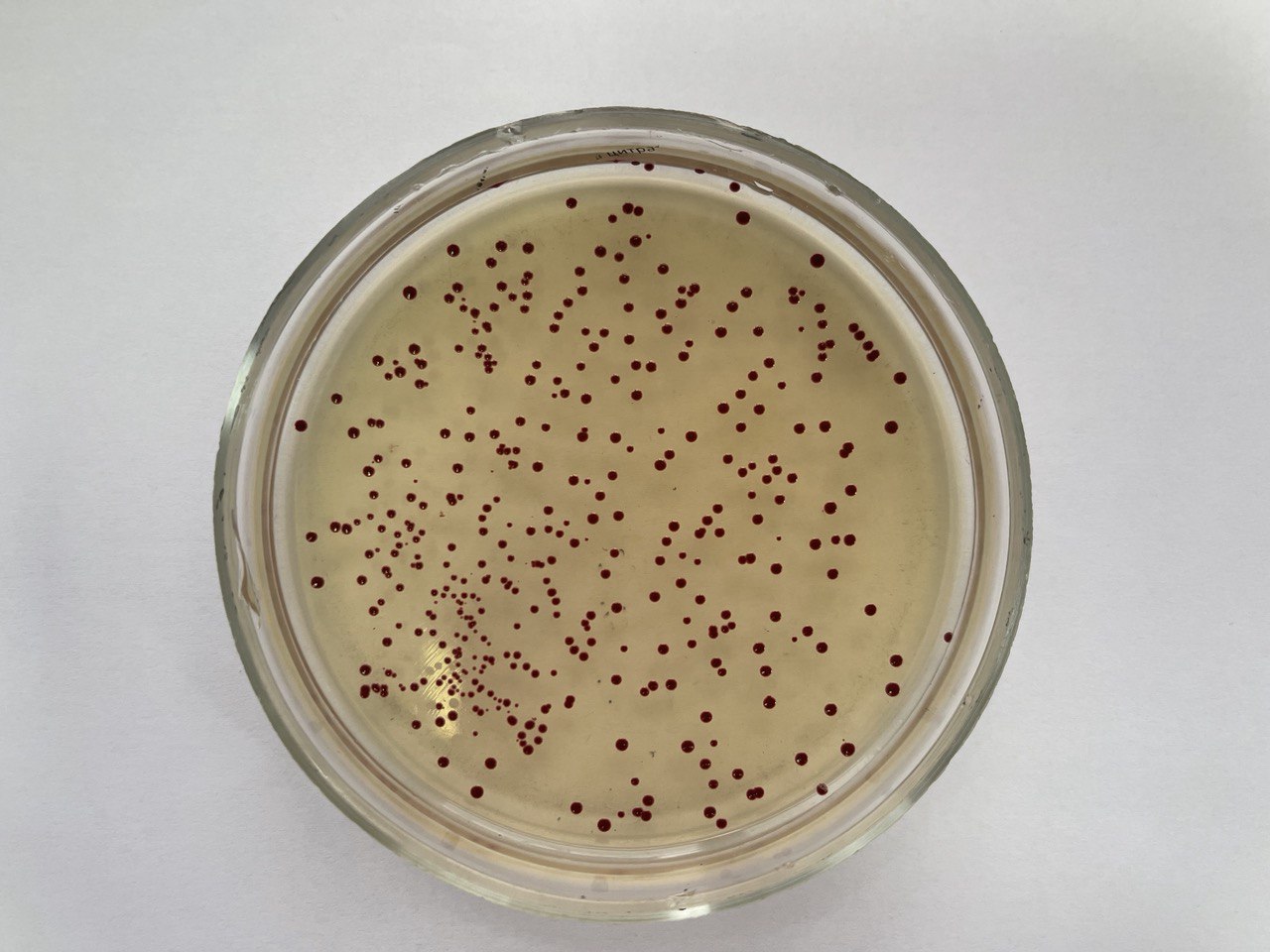
**

**Supplementary Figure 5. Bacterial Colonies Cultured on Mannitol salt agar (Biolife Italiana S.r.l., Italy).**

**
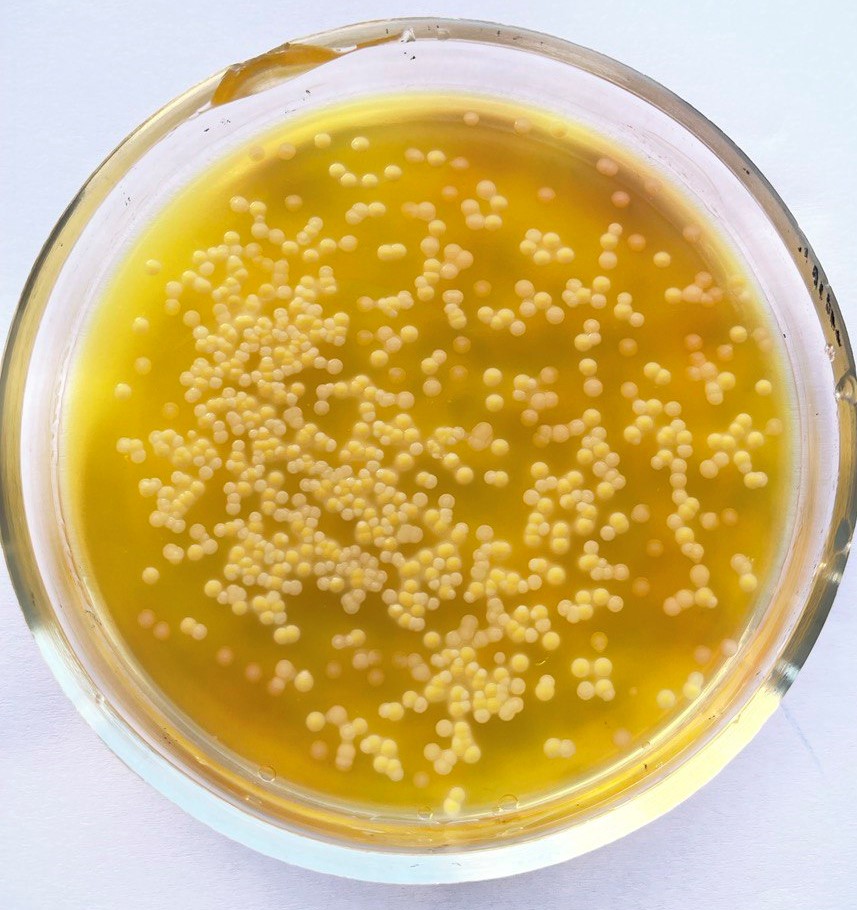
**

**Supplementary Figure 6. Yeasts Colonies Cultured on Sabouraud dextrose agar with chloramphenicol (Graco Biotech, Poland)**


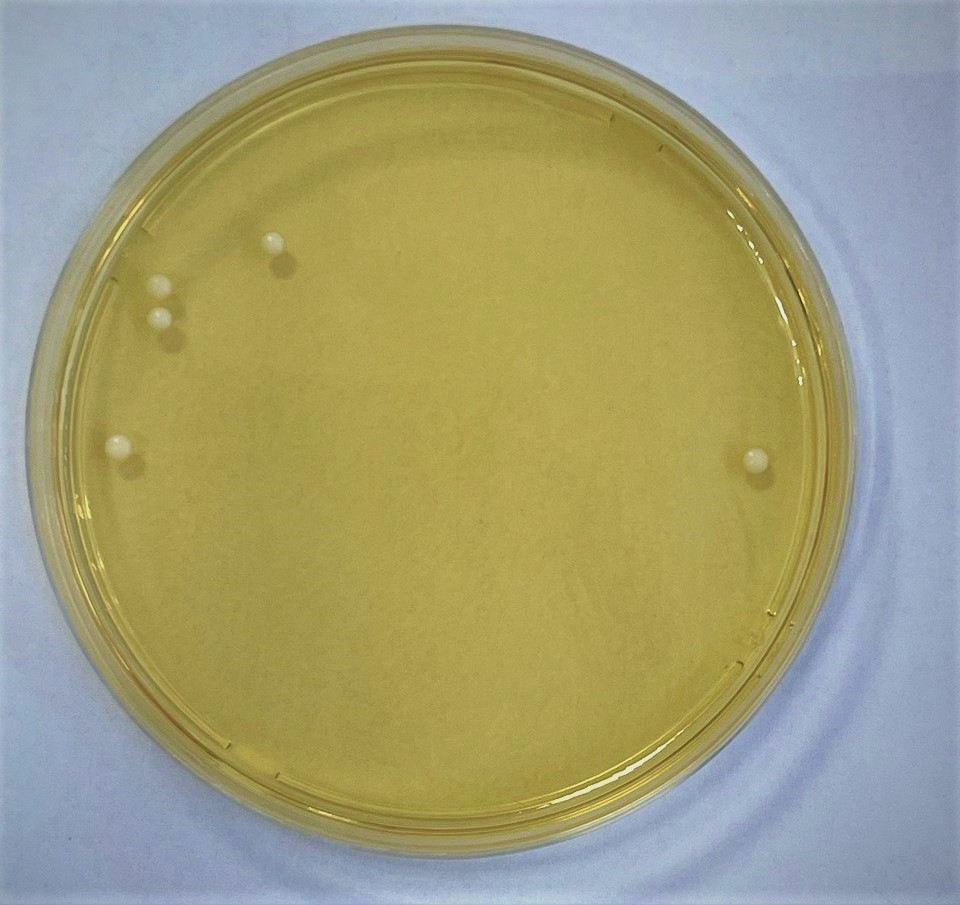

Supplement: Supplementary file 1 [file DataSheet_1.docx]
